# Usage of fMRI for pre-surgical planning in brain tumor and vascular lesion patients: Task and statistical threshold effects on language lateralization^☆^^☆☆^

**DOI:** 10.1016/j.nicl.2014.12.014

**Published:** 2014-12-24

**Authors:** Tanvi N. Nadkarni, Matthew J. Andreoli, Veena A. Nair, Peng Yin, Brittany M. Young, Bornali Kundu, Joshua Pankratz, Andrew Radtke, Ryan Holdsworth, John S. Kuo, Aaron S. Field, Mustafa K. Baskaya, Chad H. Moritz, M. Elizabeth Meyerand, Vivek Prabhakaran

**Affiliations:** a School of Medicine and Public Health, University of Wisconsin — Madison, USA; b Medical Scientist Training Program, University of Wisconsin — Madison, USA; c Department of Radiology, University of Wisconsin — Madison, USA; d Department of Medical Physics, University of Wisconsin — Madison, USA; e Department of Biomedical Engineering, University of Wisconsin — Madison, USA; f Department of Neurological Surgery, University of Wisconsin — Madison, USA

**Keywords:** fMRI, Lateralization index (LI), Thresholding, Task-specific, Surgical planning, AWG, antonym-word generation, LWG, letter-word generation, LI, lateralization index

## Abstract

**Background and purpose:**

Functional magnetic resonance imaging (fMRI) is a non-invasive pre-surgical tool used to assess localization and lateralization of language function in brain tumor and vascular lesion patients in order to guide neurosurgeons as they devise a surgical approach to treat these lesions. We investigated the effect of varying the statistical thresholds as well as the type of language tasks on functional activation patterns and language lateralization. We hypothesized that language lateralization indices (LIs) would be threshold- and task-dependent.

**Materials and methods:**

Imaging data were collected from brain tumor patients (n = 67, average age 48 years) and vascular lesion patients (n = 25, average age 43 years) who received pre-operative fMRI scanning. Both patient groups performed expressive (antonym and/or letter-word generation) and receptive (tumor patients performed text-reading; vascular lesion patients performed text-listening) language tasks. A control group (n = 25, average age 45 years) performed the letter-word generation task.

**Results:**

Brain tumor patients showed left-lateralization during the antonym-word generation and text-reading tasks at high threshold values and bilateral activation during the letter-word generation task, irrespective of the threshold values. Vascular lesion patients showed left-lateralization during the antonym and letter-word generation, and text-listening tasks at high threshold values.

**Conclusion:**

Our results suggest that the type of task and the applied statistical threshold influence LI and that the threshold effects on LI may be task-specific. Thus identifying critical functional regions and computing LIs should be conducted on an individual subject basis, using a continuum of threshold values with different tasks to provide the most accurate information for surgical planning to minimize post-operative language deficits.

## Highlights

• FMRI calculation of language lateralization is valuable for pre-surgical planning.
• Both applied statistical thresholds and task specificity affect lateralization index.
• A continuum of threshold values provides a dynamic range for presurgical planning.
• Reorganization of language function is dependent on brain pathology.
• Identification of critical language areas will optimize lesion resection (e.g. tumor).

## 1 Introduction

Functional magnetic resonance imaging (fMRI) is used to non-invasively map task-specific brain activation for pre-surgical planning (Stippich et al., 2007). These maps are generated from changes in the blood oxygen level-dependent (BOLD) signal related to neural activity (Detre and Wang, 2002). Lateralization index (LI), a measure of hemispheric dominance, quantifies information representing BOLD activation during language and cognitive tasks (Springer et al., 1999; Seghier, 2008). The degree to which functional activity lateralizes in brain tumor and vascular lesion patients is a useful guide in designing a surgical approach that minimizes post-operative neurological deficits (Vikingstad et al., 2000; Wood et al., 2011; Prabhakaran et al., 2007; Kundu et al., 2013; Sabsevitz et al., 2003). Pre-surgical maps help determine the extent to which a brain tumor or vascular lesion may be resected in patients by localizing key regions of eloquent cortex.

A number of neuroimaging studies have examined different types of language LIs and different factors that may influence the LI. A study by Ruff et al. (2008) addressed how brain tumors may modulate the LI by using a range of statistical threshold values and showed that patients demonstrate greater variability in LI during language tasks than controls (Ruff et al., 2008). Compared to strong left-lateralization in controls, brain tumor patients showed bilateral dominance at low threshold values, but left-lateralization at more stringent threshold values. The study, however, reported results on nine patients. A study by Partovi et al. (2012) used an fMRI protocol to determine language LI. Measurements were limited to Broca's (Brodmann areas 44 and 45) and Wernicke's (Brodmann area 22) areas, which are known to be primarily activated during expressive and receptive language tasks, respectively. However, other higher-order language association areas (i.e. supramarginal, middle and inferior frontal gyrus) may be involved with language function, but few studies have examined reorganization in these regions following a lesion (Partovi et al., 2012; Rypma et al., 2006; Rypma et al., 2009; Vigneau et al., 2006).

Janecek et al. (2013) investigated language lateralization in a large group of patients to evaluate the clinical effectiveness of fMRI compared to WADA testing. Upon utilization of fMRI and WADA protocols, the scientists found that 80% of normal subjects were left lateralized in language function. Overall, Janecek et al. (2013) determined that the discordance rates between LI obtained from fMRI and WADA protocols were quite small, suggesting that the utilization of fMRI for pre-surgical planning is just as effective of a tool as the WADA testing. Janecek et al. (2013) found that the degree of bilateral and rightward shift of language lateralization was more clearly evident in the fMRI results. However, it cannot be concluded that the altered language lateralization seen in the fMRI results are more or less accurate compared to the WADA results. The Janecek et al. (2013) study suggests that fMRI may have increased sensitivity to language processing in the right hemisphere compared to the WADA test. The previous study stated that the discordance rates could have been influenced by the types of tasks used in fMRI (Janecek et al., 2013). Utilizing fMRI in our study, we investigated the impact of varying the thresholds after performance of different task types on the patients' patterns of language lateralization.

A study by Friederici et al. (2000) showed that language lateralization patterns differ based on the type of task performed. Additionally, Ruff et al. (2008) proposed that language lateralization is dependent on the statistical threshold utilized (Ruff et al., 2008; Friederici et al., 2000). Studies have shown that the presence of brain tumors and vascular lesions affects neurological function in a language network (Vikingstad et al., 2000; Prabhakaran et al., 2007; Sabsevitz et al., 2003; Bookheimer, 2007). However, a large number of these studies have focused on patients with brain tumors. Few studies have examined the influence of a lesion on language reorganization patterns in patients with AVMs and cavernomas (Lee et al., 2010). Thus the analyses were repeated for both tumor patients and a collection of vascular lesion patients.

Wellmer et al. (2009) investigated the impact of lesions on language lateralization through fMRI and WADA test protocols and determined that a variable fMRI language lateralization is observed in vascular lesion patients (Wellmer et al., 2009). The study categorized average LI measurements between 0.3 and 1.0 as unilateral dominant, while the current study utilizes the ranges of +0.2 < LI < +1.0 (left lateralized), −0.2 < LI < +0.2 (bilateral), and −0.2 < LI < −1.0 (right lateralized) as determined by Springer et al. (1999) to evaluate the strength of lateralization in patients (Springer et al., 1999; Wellmer et al., 2009). Categorization of the LI values helps summarize the general effect that brain tumors or vascular lesions may have on the language network.

Studies have shown that a fixed threshold may be insufficient to detect differences in voxel activation between hemispheres, resulting in inaccurate calculation of the LI (Sabsevitz et al., 2003; Pillai and Zaca, 2011). Thus the present study investigated the effect of varying threshold values on the ability to detect pseudoreorganization of task-specific language function, as measured by average LI, in a large population of brain tumor (n = 67) and vascular lesion patients (n = 25). We used a network of ROIs that incorporated higher-order language association areas and the two dominant language areas of the brain to determine average hemispheric LI and activation patterns (Partovi et al., 2012; Vigneau et al., 2006). Lastly, we report how LI varies with task type and the level of statistical thresholding (Sabsevitz et al., 2003). We propose that threshold-dependence and task selection are important factors to consider in identifying subject-specific lateralization patterns and localizing critical areas of activation.

## 2 Methods

### 2.1 Subjects

Anatomical and functional images were derived from a database (between 2004 and 2011) of patients who underwent an fMRI pre-surgical planning protocol. We retrospectively selected 92 right-handed patients diagnosed with either left-hemispheric brain tumors or vascular lesions who received a pre-operative fMRI at the University of Wisconsin — Madison, and had provided written informed consent for their data to be used for applicable research purposes. We selected only right-handed patients to reduce the confounding effects due to handedness, with the notion that right-handed individuals display left-lateralized language activation patterns (Knecht et al., 2000). The analysis reported includes data from 67 brain tumor patients (43 males, 24 females) and 25 vascular lesion patients (9 males, 16 females). Also, 25 right-handed healthy controls (13 males, 12 females) performed the letter-word generation task and were used as a comparison to patient data. Patient demographics are reported in Tables 1, 2, and 3. There was no significant difference in age between the groups; however, age may influence language lateralization. Szaflarski et al. (2006) concluded that there is a slow decrease in dominant hemisphere lateralization in adults as they age (Szaflarski et al., 2006). The present study utilized data including young to older adults and, therefore, may show age-related effects on LI, but this was controlled for between the three groups. Gender differences were significant among tumor patients and among vascular lesion patients (p < 0.004). Preliminary evaluation was conducted to investigate the effect that gender has on thresholded task-specific LI (Table 4). The protocol was approved by the University of Wisconsin — Madison Health Sciences institutional review board.

### 2.2 fMRI image acquisition

FMRI scanning was conducted using a 1.5 or 3 T commercial MR imaging scanner (Sigma General Electric Healthcare, Milwaukee, Wisconsin) with high-speed gradient capabilities. Technical scanner parameters include: FOV, 24 cm; matrix 64 × 64; TR, 2000 ms; TE, 40 ms (for 1.5 T) or 27 ms (for 3T); flip angle, 85° (for 1.5 T) or 75° (for 3 T); 6-mm coronal plane sections (for 1.5 T) or 5-mm axial plane sections (for 3 T). Additional high-resolution anatomical scans, including 3D volumetric T1- and T2-weighted sequences were acquired as part of the pre-operative assessment.

### 2.3 fMRI task paradigms

Patients were instructed to perform expressive and/or receptive language tasks based on individual medical conditions to help the neurosurgical team evaluate specific language function. All tasks followed a block design paradigm, which consisted of subjects alternating between 20 s of rest (blank screen or non-relevant information) and 20 s of task (activation state) for five cycles approximating three and a half minutes total.

#### 2.3.1 Expressive language tasks

The expressive language tasks were designed to primarily activate Broca's area (inferior frontal gyri) and secondarily activate Wernicke's area (posterior superior temporal gyri) and surrounding higher order language association areas, such as the supramarginal and middle frontal gyri. The two expressive language tasks were the antonym-word generation (AWG) (tumor, n = 57; vascular lesion, n = 23) and the letter-word generation (LWG) (tumor, n = 42; vascular lesion, n = 10). Due to the retrospective nature of the study, each patient performed a different number of language tasks. Each tumor and vascular lesion patient performed one or both of the expressive language tasks (AWG and/or LWG). During the AWG task, subjects covertly produced a list of antonyms to the words on the screen. During the LWG task, subjects covertly generated a list of words beginning with the letters on the screen. Comparison between tumor and vascular lesion patient performance on expressive tasks was not conducted because it was outside the parameters of the present study's focus and the subjects within each patient group did not perform both tasks equally.

#### 2.3.2 Receptive language tasks

The receptive language tasks (text-reading and text-listening tasks) were designed to primarily activate Wernicke's area and secondarily activate Broca's area and surrounding higher order language association areas. Each patient group performed a different receptive language task. Of the tumor patients who performed a receptive language task, they performed a text-reading task (n = 25). During this task, patients covertly read a block of text for comprehension. Of the vascular lesion patients who performed a receptive language task, they performed the text-listening task (n = 12). During this task, patients listened to a narrated text for comprehension via in-ear sound isolating headphones. Comparison between tumor and vascular lesion patient performance on receptive tasks was not conducted because it was outside the parameters of the present study's focus and each patient group performed a different receptive language task.

### 2.4 Data analysis

The fMRI data were processed using AFNI (Analysis of Functional NeuroImages) available at http://afni.nimh.nih.gov/afni/. Preprocessing steps included dropping the first three volumes, motion correction, spatial smoothing (6 mm FWHM), and spatial normalization to 3 × 3 × 3 mm. AFNI's 3dDeconvolve program was used to perform voxel-wise regression analysis to generate thresholded t-maps. The six motion parameters (x, y, z, pitch, roll, yaw) were regressed out in the analysis. 5 mm regions of interest (ROIs) were created based off of MNI (Montreal Neurological Institute) coordinates reported in a meta-analysis of language areas including Brodmann areas 44 and 45 (Broca's area) and areas 22, 39, and 40 (Wernicke's area) and other associated motor and language areas described in Vigneau et al. (2006) (Vigneau et al., 2006). We obtained left hemisphere language areas based on these coordinates, generated homologous ROIs in the right hemisphere, and computed LI for each predefined network ROI-pair. The same protocol for analysis was applied to both tumor and vascular lesion data.

### 2.5 Calculation of lateralization index

The following equation was used to determine the LI values: LI = (VL − VR) ∕ (VL + VR), where VL and VR correspond to the number of active voxels in the left and right hemispheres, respectively (Springer et al., 1999). The scale used to categorize LI was as follows: +0.2 < LI < +1.0 (left-lateralized), −0.2 ≤ LI ≤ +0.2 (bilateral), and −0.2 < LI < −1.0 (right-lateralized) (Springer et al., 1999). These values were calculated for both hemispheres at four different thresholds (t < 2, t < 2.67, t < 3.5, t < 4), which correspond to the following p values (p < 0.05, p < 0.01, p < 0.001 and p < 0.0001), respectively.

### 2.6 Statistical analysis

To determine if there was an effect of threshold value between the patient groups and the control group, we compared each patient group's average LI at all t-values with the control using an independent two-sample t-test. We compared the difference between LIs at all pairs of t-values (e.g. t < 2 vs. t < 2.67, t < 2 vs. t < 3.5) to specifically analyze the effect of t-values on different tasks within each patient group. Bonferroni correction was used to correct for multiple comparisons. A two-sample t-test was performed to generate group activation maps.

## 3 Results

**Fig. 1:**
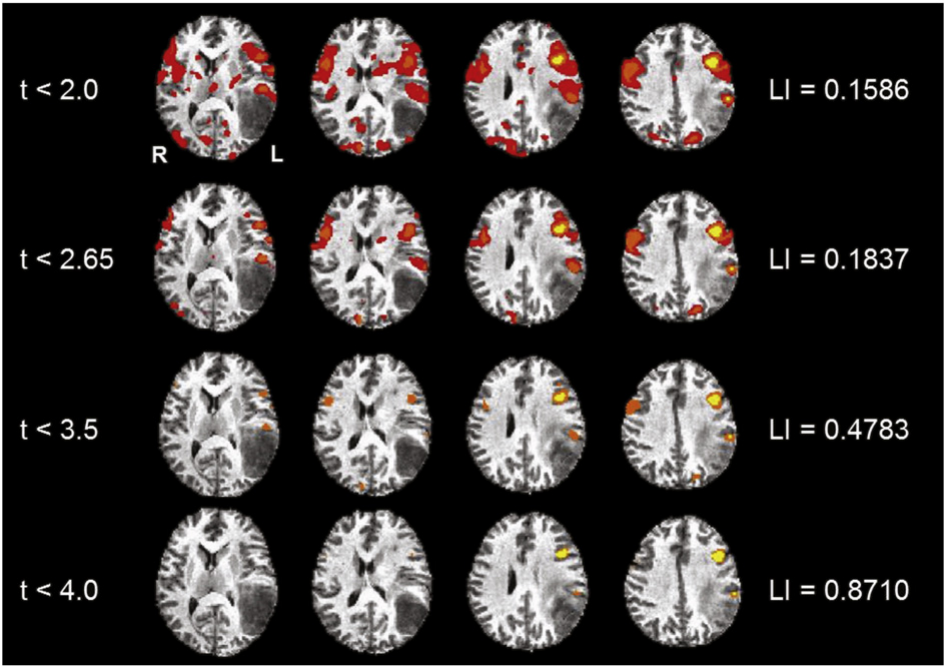
An example of variable thresholding in a tumor patient. These images are of a tumor patient after performing the receptive language, text-reading task showing the general trend we observed for most of the tasks of shifting from a bilateral pattern of language activity to a left lateral pattern as the threshold becomes more stringent. The images follow a radiological convention with left hemisphere (L) on the right and right hemisphere (R) on the left. Talairach functional maps of all the patients are averaged together and overlaid onto the MNI 152 standard anatomical template.

**Fig. 2:**
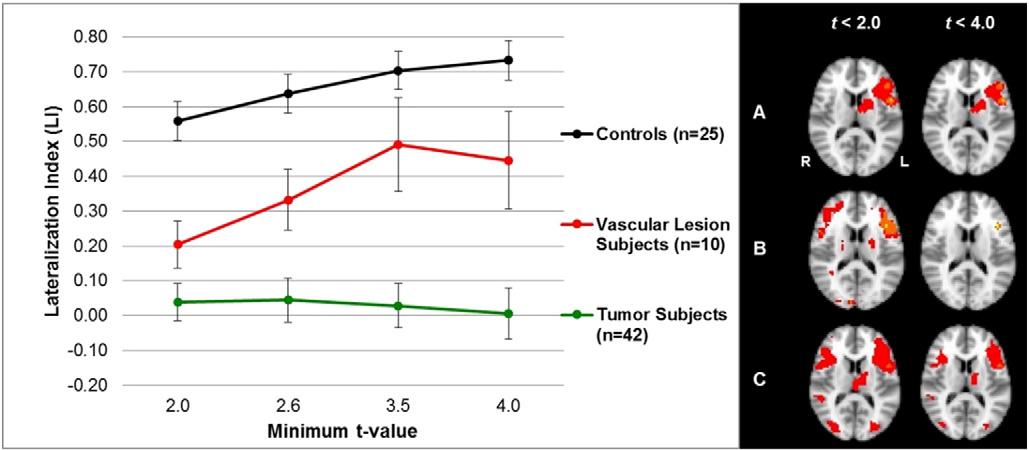
Average hemispheric LIs (left) and group activation (right) for letter-word generation task in the control, tumor, and vascular lesion patient groups. Left: the average LIs for tumor patients (n = 42), vascular lesion patients (n = 10), and controls (n = 25), from the letter-word generation task, are shown at different threshold values for the hemispheric mask. Tumor patients show bilateral dominance regardless of t-value. Tumor patient LIs are significantly different from control LIs at each threshold (ps < 0.0125, Table 5). Vascular lesion patients show bilateral dominance at t < 2.0 and left lateralization at t < 4.0. Vascular lesion patient LIs are significantly different from control LIs at at t < 2.0 (p < 0.0125, Table 5). The control group maintains left lateralization despite variable threshold effects. Right: images display the group activation maps at low threshold values (ts < 2.0) and at higher threshold values (ts < 4.0). The control subjects (A) show average left language lateralization, the vascular lesion patients (B) show average bilateral and left-lateral dominance, and the tumor patients (C) show average bilateral dominance during the letter word generation task.

The predominant activation pattern we observed in brain tumor and vascular lesion patients indicated that as the threshold was increased from a low to a high threshold, the average LI became more left-lateralized, except for the brain tumor patient group performing the LWG task (Figs. 1 and 2). The control group showed left-lateralization in the inferior, middle, and superior frontal gyri across all threshold values during the LWG task. In contrast, the brain tumor patient group showed bilateral activity in the inferior, middle, and superior frontal gyri, and supramarginal and inferior parietal gyri across all threshold values. The vascular lesion patient group showed bilateral activity in the inferior, middle, and medial frontal gyri, inferior parietal gyri, and superior temporal gyri at a low threshold value (t < 2.0), but showed robust left hemispheric activity in the inferior and middle frontal gyri at high threshold values (ts < 3.5) (Fig. 2). There was significantly more bilateral activity in the tumor patient group compared to the control group across all t-values. The vascular lesion patient group showed a significantly bilateral LI only at t < 2.0 compared to the control group (Fig. 2 and Table 5).

### 3.1 Expressive tasks

**Fig. 3:**
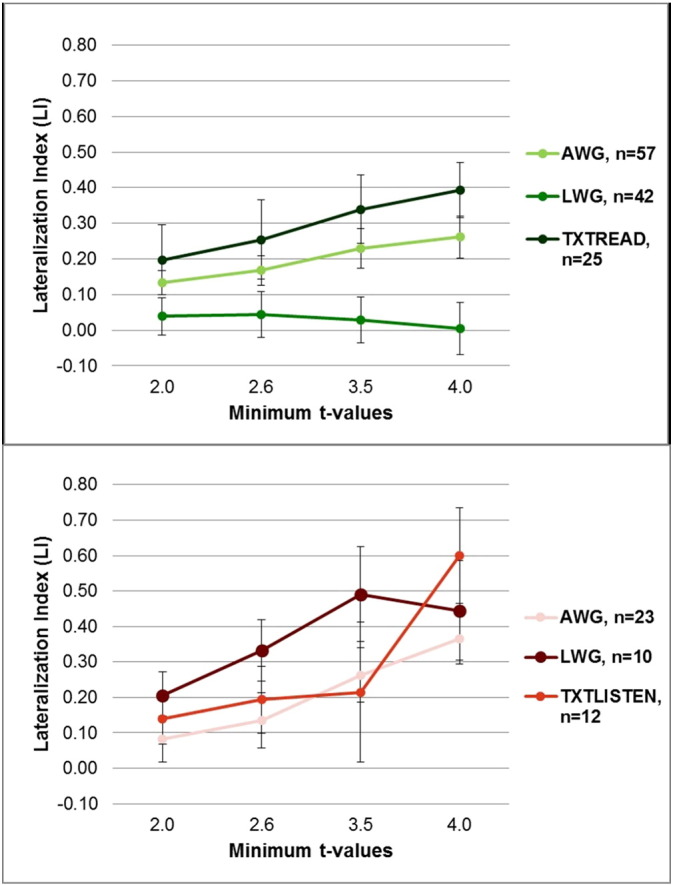
Average hemispheric LIs in tumor (top) and vascular lesion (bottom) patients at different threshold values. Top: tumor patients' average LIs for antonym-word generation task (AWG) (n = 57), letter-word generation task (LWG) (n = 42), and text-reading task (TXTREAD) (n = 25) are shown for four t-values (t < 2.0, t < 2.6, t < 3.5, t < 4.0). Bottom: vascular lesion patients' average LIs for antonym-word generation task (AWG) (n = 23), letter-word generation task (LWG) (n = 10), and text-listening task (TXTLISTEN) (n = 13) are shown for four t-values (t < 2.0, t < 2.6, t < 3.5, t < 4.0).

**Fig. 4:**
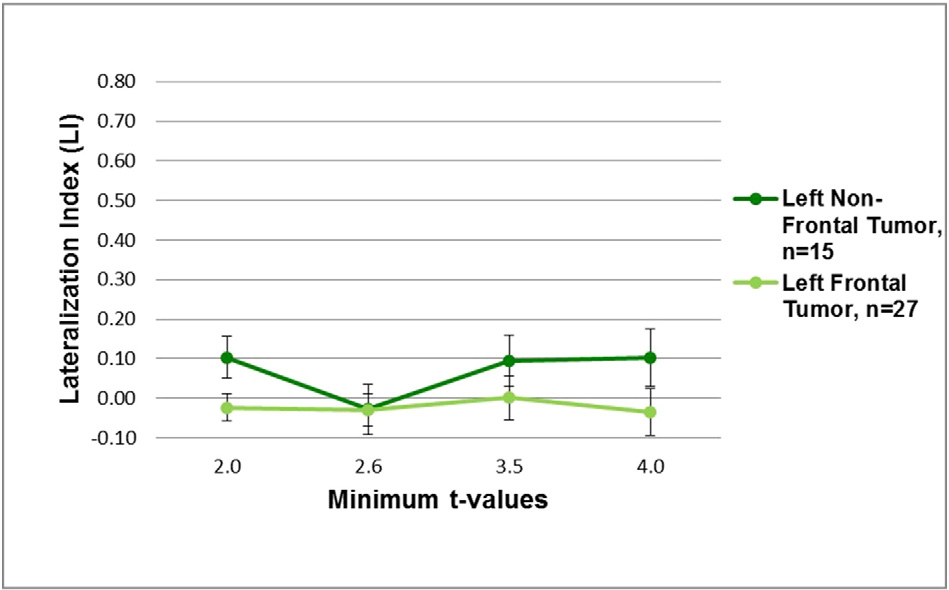
Average hemispheric LIs in left frontal tumor patients (n = 27) and left non-frontal tumor patients (n = 15) at different threshold values (t < 2.0, t < 2.6, t < 3.5, t < 4.0) performing the letter-word generation (LWG) task.

The average LIs for the tumor patient group that performed the AWG task showed bilateral activation at low threshold values (ts < 2.6), but left-lateral activation at high threshold values (ts < 4.0) (Table 6 and Fig. 3, top). In contrast, despite varying the threshold, bilateral activation was maintained during the LWG task (Fig. 3, top). Also all of our patients had brain tumors in the left hemisphere with no differences between frontal and non-frontal tumor patients with both demonstrating bilateral activation during the LWG task (Fig. 4).

The average LIs for the vascular lesion patient group that performed the AWG task showed bilateral activation at low threshold values (ts < 2.6) and left-lateral activation at high threshold values (ts < 4.0) (Fig. 3, bottom). Similarly, LIs derived from the LWG task indicated bilateral activation at a low threshold value (t < 2.0) and left-lateral activation at high threshold values (ts < 4.0); however, this effect was not statistically significant (Table 6 and Fig. 3, bottom).

### 3.2 Receptive tasks

The average LI of the tumor patients performing the text-reading task showed bilateral activation at a low threshold value (t < 2.0) and left-lateral activation at higher threshold values (ts < 4.0) (Table 6 and Fig. 3, top).

Vascular lesion patients performed the text-listening task. This task demonstrated the same qualitative effects as tumor patients performing the text-reading task in that the average LI was bilateral at low threshold values and left-lateralized at high threshold values (Fig. 3, bottom); however, this effect was not statistically significant (Table 6).

### 3.3 Gender differences

Gender differences may influence language processing in this patient population. Calculating average LI within each patient group, based on gender, we found bilateral activity across thresholds in the female tumor patients performing the AWG task (n = 23) and the LWG task (n = 16), but differences in male tumor patients with more left lateralization at higher thresholds in the AWG task (n = 34) and bilateral activity in the LWG task (n = 26). This suggests that in brain tumor patients there may be a gender effect with females showing greater bilateral involvement irrespective of the expressive task. Vascular lesion patients lacked sufficient male numbers to assess gender effects in the present study. However, the female vascular lesion patients showed predominantly a left-lateralized activity at higher thresholds irrespective of task type in contrast to female brain tumor patients who showed bilateral activity across all thresholds (Table 4 and Fig. 3). This suggests that lesion type as well as gender may influence language lateralization.

### 3.4 Result summary

At a more stringent threshold value along a threshold continuum, we generally saw a shift from bi-lateralization to left-lateralization (result summary Table 7), with the exception of the tumor patient group and control subjects performing the LWG task. Thresholding had no effect on the tumor patient group's average LI for this task; the subjects showed bilateral activation regardless of threshold value. Thresholding had no significant effect on the control group LI; this group showed left-lateralization despite the variable threshold. In summary, average LI values in vascular lesion patients are sensitive to varying the threshold in this study; in contrast, average LI values in tumor patients demonstrated sensitivity to varying the threshold and task specificity in this study.

## 4 Discussion

This study aimed to utilize an fMRI-based LI to quantify how sensitive language dominance was to the threshold applied to BOLD activations and the specific task type in tumor and vascular lesion patients. Within the brain tumor patient group, varying threshold values affected LI derived from the AWG (expressive) task and the text-reading (receptive) task. During these tasks, on average, patients showed a shift from bilateral to left-lateralization as the t-values became more stringent. Our results are consistent with previous studies suggesting that LI during the AWG and text-reading tasks performed by brain tumor patients is sensitive to thresholding effects (Zacà et al., 2012; Suarez et al., 2009). LI derived from the LWG task, however, was not affected by threshold variance, yielding bilateral LIs across all t-values. This suggests that the average LI in tumor patients is sensitive to both task type and thresholding effects.

Variability in activation observed in language associated cortical regions during different tasks performed by the patient groups, provides evidence that independent pathways may be activated to complete the task. Robust activity was seen in the inferior and middle frontal gyri during expressive tasks. Activity dominated in the inferior parietal/superior temporal gyri during receptive tasks. Expressive language tasks rely on semantic memory and word generation (Friederici et al., 2000). In contrast, receptive language tasks rely on visual and auditory comprehension (Friederici et al., 2000). The difference observed in the two expressive language tasks performed by brain tumor patients may be explained by a comprehension component that is common to the AWG (expressive) task and the text-reading (receptive) task, but absent from the LWG (expressive) task. The AWG task requires the subject to comprehend the word presented before generating antonyms. Therefore, we believe there may be a comprehension component that prompts the activation of a separate language pathway further explaining the difference between the performances of the two expressive language tasks in brain tumor patients. Within the vascular lesion patient group, varying threshold values affected LI derived from both the expressive and receptive tasks. These patients showed bilateral activation at low threshold values, but left-lateral activation at higher threshold values. These results suggest that thresholding has an effect on the LI measurements in vascular lesion patients. Average LI values were similar across tasks, suggesting that language lateralization in vascular lesion patients is not dependent on task type.

Variation of the activation patterns due to varying threshold values provides evidence that different tasks utilize different language networks (Sabsevitz et al., 2003; Friederici et al., 2000). Furthermore, the differences in our results between brain tumor and vascular lesion patients suggest that pathology differentially affects the BOLD response, which influences the activation patterns. The LWG (expressive) task primarily activates Broca's area in healthy controls. In contrast, the activation map for the LWG task performed by brain tumor patients showed bilateral activity in the frontal, parietal and temporal regions. In addition, since the LWG task activates predominantly the left frontal regions of the brain in the normal subjects, we evaluated whether the majority of our patients that had left frontal tumors may have contributed to the bilateral activity seen across threshold values during the LWG task. Further investigation of the tumor patients that performed the LWG task revealed that patients with a left frontal tumor (n = 27) and patients without a left frontal tumor (n = 15) maintained bilateral activity at each threshold value. Given that all of our patients had left hemisphere tumors, this may have influenced left frontal as well as other left hemisphere language areas (parietal, temporal) in recruiting right hemisphere areas. Vascular lesion patients only showed bilateral activity at low threshold values for both expressive tasks (AWG and LWG), suggesting that the non-affected right hemisphere was recruited but became left-lateralized at higher threshold values. Compared to the control group, both brain tumor and vascular lesion patient groups demonstrated greater bilateral dominance. So lesion type also plays a role in the ability to recruit right hemisphere areas. These results are consistent with previous studies that report activation in homologous language associated regions of the unaffected right hemisphere indicating pseudoreorganization in brain tumor and vascular lesion patients compared to controls (Prabhakaran et al., 2007; Partovi et al., 2012; Lee et al., 2010). We also found differences in gender with greater bilateral activity irrespective of the expressive task in female brain tumor patients. The female vascular lesion patients showed predominantly left-lateralized activity at higher thresholds irrespective of task type, in contrast to female brain tumor patients who showed bilateral activity across all thresholds. This suggests that lesion type as well as gender may influence language lateralization. Overall, different network pathways may have undergone pseudoreorganization of language processing directed towards the unaffected right hemisphere in vascular lesion and brain tumor patients, which may be the result of compensation or reduced neural efficiency (Prabhakaran et al., 2007; Partovi et al., 2012; Janecek et al., 2013; Wellmer et al., 2009).

The application of a variable statistical threshold influences the presumed hemispheric dominance of language, as determined by LI, for both tumor and vascular lesion patients. Our findings indicate that LI in tumor patients is also influenced by the task performed. Clinically, these findings indicate that during pre-surgical planning, data from different language tasks and thresholds should be considered cumulatively to identify regions that may be critical for language function. Niskanen et al. (2012) investigated fMRI data from an array of language tasks to characterize language dominance for clinical purposes. Consistent with our study, Niskanen et al. (2012) found that auditory and visual tasks that activate a range of language regions (i.e. word generation and sentence comprehension tasks) are effective for measuring language dominance (Niskanen et al., 2012). Activation maps derived from a range of threshold values and tasks can help neurosurgeons devise an optimal approach for resection while sparing eloquent cortex important for language function.

Our results show that LI calculated at t < 4.0 is a prime indicator of language hemispheric dominance, as it incorporates robust (p < 0.0001) activation. FMRI activity at threshold values more stringent than t < 4.0 provides less activation, resulting in less reliable LI calculations. Therefore, an appropriate range of threshold values should be utilized to obtain accurate data and improve post-surgical outcomes. The measurements of average LI at different threshold values show that patients may show bilateral activity at low t-values, but only left hemispheric activity may be present at more stringent t-values. Measuring LI at a fixed threshold value provides an inaccurate representation of the cortex involved in language activation. A fixed threshold value may lead clinicians to inaccurately perceive the right hemisphere to be equally as dominant as the left hemisphere in language function, prompting imprecise surgical resection. A variable statistical threshold is a valuable tool to identify consistent robust bilateral activity, as seen in the brain tumor patient LWG task data. This tool may maximize the precision, optimize the degree of resection, and minimize post-operative neurological deficits.

There are several limitations to this study. Foremost, there was no task-equivalent control group for the AWG and text-reading/listening tasks. In addition, due to the retrospective nature of this study, each subject did not perform an equal number of language tasks. Therefore, some patients within each group performed one expressive language task, while others performed both expressive language tasks. Among the patients that performed a receptive language task, the tumor patients performed a text-reading task, while vascular lesion patients performed a text-listening task. Due to this limitation, each subject within the patient groups performed a different number of tests compared to each other as well as the subjects in the control group. Therefore, the patients within each group had a differing amount of language task practice/experience compared to others, which may have an influence on LI values. Additionally, the degree of language-related deficits (e.g. aphasia) present while performing the different tasks was not considered, but is important when deciding the functional significance of an activated area on fMRI. Also majority of these patients did not receive WADA testing, which is not the clinical practice for brain tumor and vascular lesion patients at our institution. So we are unable to compare WADA and fMRI results in our study. Future studies should undertake WADA testing to compare with fMRI lateralization or LI. Besides LI, individual patient performance on the tasks was not incorporated into our analysis due to the covert nature of their performance in order to minimize motion in the MRI scanner. Each patient group did not perform the same receptive task and gender was not appropriately matched between groups. Finally, we were not able to stratify data by lesion type, size, or location due to the sample sizes. Future studies with larger sample sizes could evaluate the influence of these factors on language lateralization.

## 5 Conclusion

Language lateralization is dependent on the statistical threshold applied to task-derived fMRI data in tumor and vascular lesion patients. We observed that as the applied threshold increased from low to high, the average LI became more left-lateralized, with the exception of a task performed by the tumor patient group. Pre-surgical teams should consider the lesion pathology and task specificity along a continuum of statistical threshold values to evaluate language function when assessing fMRI results. Application of this protocol to different language tasks may further improve post-operative outcomes in tumor and vascular lesion patients.

## Author contributions

Both Nadkarni TN and Andreoli MJ contributed equally and are co-principal authors on this manuscript.

Andreoli MJ and Nadkarni TN were involved in the data analysis, interpretation, literature search, and writing of the manuscript.

Nair VA was involved in the data collection, analysis, interpretation, literature search, and writing of the manuscript.

Yin P was involved in the data analysis.

Young BM was involved in the data collection and analysis.

Kundu B was involved in the review and writing of the manuscript.

Pankratz J was involved in the data analysis.

Radtke A was involved in the data analysis.

Kuo JS was involved in patient referrals.

Field AS was involved in study design.

Holdsworth R was involved in data collection.

Baskaya MK was involved in patient referrals.

Moritz CH was involved in data collection.

Meyerand ME was involved in study design.

Prabhakaran V was lead principal investigator on the study and was involved in study design and data collection.

## Conflict of interest

The authors declare no conflict of interest.

## Figures and Tables

**Table 1:** Patient demographics.

Characteristics of patients	Tumor patients (n = 67)	Vascular lesion patients (n = 25)	Controls (n = 25)	p-Value (p < 0.05)
Males (%)	64%	36%	52%	T vs. V: p = 0.004
Females (%)	36%	64%	48%
Age range [mean] (years)	21–82 [48]	20–72 [43]	20–75 [45]	NS
Number of patients per task				
Antonym-word generation	57	23	0	
Letter-word generation	42	10	25	
Text-reading	25	0	0	
Text-listening	0	12	0	

**Table 2:** Tumor patient lesion demographics.

Tumor	Number of patients
Type	
Astrocytoma	22
Oligodendroglioma	11
Glioblastoma multiforme	9
Meningioma	4
Metastatic	4
Oligoastrocytoma	3
Fibrillary astrocytoma	2
DNET	2
Anaplastic astrocytoma	1
Hemangiopericytoma	1
Benign cyst	1
Undefined	2
Other	5
Grade	
1	7
2	20
3	10
4/metastasis	24
Benign	4
Undefined	2
Location	
Left frontal	35
Left temporal	12
Left parietal	5
Left insula	3
Left frontal+	8
Left parietal+	3
Left temporal+	1

**Table 3:** Vascular lesion patient lesion demographics.

Vascular lesion	Number of patients
Type	
AVM	13
Cavernoma	8
Hemorrhage (no underlying lesion found)	3
Aneurysm	1
Location	
Left frontal	3
Left temporal	9
Left parietal	2
Left insula	1
Left frontotemporal	1
Left frontoparietal	5
Left temporoparietal	2
Left parietal-occipital	2

**Table 4:** Average LI by gender.

Task	Gender/lesion	n	t < 2	t < 2.67	t < 3.5	t < 4
LWG	Male tumor	26	BL	BL	BL	BL
Female tumor	16	BL	BL	BL	BL
Male vascular lesion	3	BL	BL	LL	LL
Female vascular lesion	7	LL	LL	LL	LL
AWG	Male tumor	34	BL	LL	LL	LL
Female tumor	23	BL	BL	BL^a^	BL^a^
Male vascular lesion	8	BL	BL	LL	LL
Female vascular lesion	15	BL	BL	LL	LL
TXTREAD	Male tumor	13	BL	BL	LL	LL
Female tumor	12	BL	BL	BL	LL
TXTLISTEN	Male vascular lesion	2	BL	BL	RL^a^	BL^a^
Female vascular lesion	10	BL	LL	LL	LL

BL = bilateral, LL = left-lateral, RL = right-lateral.

a Pattern deviates from gender non-specific population averages.

**Table 5:** Patient LI versus control LI.

	Controls vs. tumor p-values (df) = 65	Controls vs vascular lesion p-values (df) = 34
t < 2.0	2.97E−09^a^	0.002428^a^
t < 2.6	4.07E−10^a^	0.013949
t < 3.5	8.88E−10^a^	0.142843
t < 4.0	3.15E−09^a^	0.061795

a Statistically significant at p < 0.0125, corrected.

**Table 6:** LI comparison at different threshold values within patient group.

	Tumor p-values			Vascular lesion p-values		
	Antonym (df) = 112	Letter (df) = 82	Text-read (df) = 48	Antonym (df) = 44	Letter (df) = 18	Text-listen (df) = 22
t < 2.0 vs. t < 2.6	0.017315	0.261239	0.982891	0.171803	0.261758	0.364700
t < 2.0 vs. t < 3.5	0.002512^a^	0.785271	0.026236	0.001628^a^	0.072703	0.625992
t < 2.0 vs. t < 4.0	0.001654^a^	0.46902	0.003302^a^	0.000018^a^	0.142270	0.011144
t < 2.6 vs. t < 3.5	0.014164	0.59038	0.004038^a^	0.000156^a^	0.334689	0.859786
t < 2.6 vs. t < 4.0	0.002848^a^	0.294369	0.012397	0.000074^a^	0.507890	0.017450
t < 3.5 vs. t < 4.0	0.015845	0.142492	0.094924	0.006476^a^	0.820798	0.175559

a Statistically significant at p < 0.008, corrected.

**Table 7:** Change in lateralization summary.

	LWG	AWG	Text-reading	Text-listening
Tumor	B (no change)	B → LL	B → LL	N/A
Vascular lesion	B → LL	B → LL	N/A	B → LL
Control	LL (no change)	N/A	N/A	N/A

B = bilateralization; LL = left-lateralization; LWG = letter-word generation; AWG = antonym-word generation.
